# Supplementary material for: Structural insights into regulation of TRPM7 divalent cation uptake by the small GTPase ARL15
Source: bioRxiv. 2023 Jan 20:2023.01.19.524765. Preprint. [Version 1] doi: 10.1101/2023.01.19.524765 (PMC9882303; doi:10.1101/2023.01.19.524765)
Supplement: Supplement 1 [file NIHPP2023.01.19.524765v1-supplement-1.pdf]

bioRxiv preprint doi: https://doi.org/10.1101/2023.01.19.524765; this version posted January 20, 2023. The copyright holder for this preprint (which was not certified by peer review) is the author/funder, who has granted bioRxiv a license to display the preprint in perpetuity. It is made available under aCC-BY 4.0 International license.

Supplemental materials for

Structural insights into regulation of TRPM7 divalent cation uptake by the small GTPase ARL15

Luba Mahbub[1,2,‡], Guennadi Kozlov[1,2,‡], Pengyu Zong[3], Sandra Tetteh[4], Thushara Nethramangalath[4], Caroline Knorn[1,2], Jianning Jiang[1,2], Ashkan Shahsavan[1,2], Emma Lee[1,2], Lixia Yue[3], Loren W. Runnels[4], Kalle Gehring[1,2,*]

[1]Department of Biochemistry, McGill University, Montréal, Canada

[2]*Centre de recherche en biologie structurale*, McGill University, Montréal, Canada

[3]Dept. of Cell Biology. UCONN Health Center, Farmington, Connecticut, United States

[4]Rutgers-Robert Wood Johnson Medical School, Piscataway, New Jersey, United States

bioRxiv preprint doi: https://doi.org/10.1101/2023.01.19.524765; this version posted January 20, 2023. The copyright holder for this preprint (which was not certified by peer review) is the author/funder, who has granted bioRxiv a license to display the preprint in perpetuity. It is made available under aCC-BY 4.0 International license.

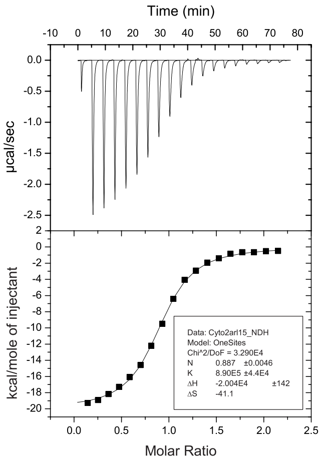

30 μM CNNM2 429-817
300 μM ARL15 32-197

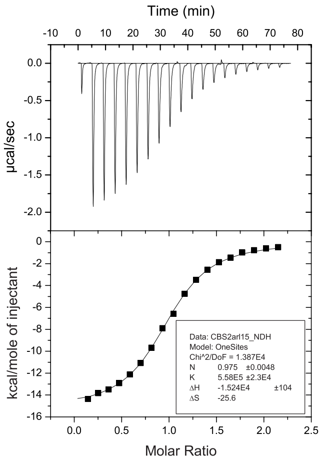

30 μM CNNM2 429-584
300 μM ARL15 32-197

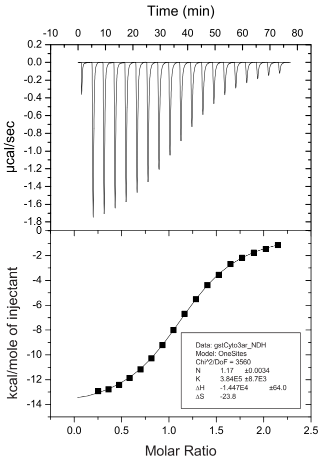

30 μM GST-CNNM3 299-658
300 μM ARL15 32-197

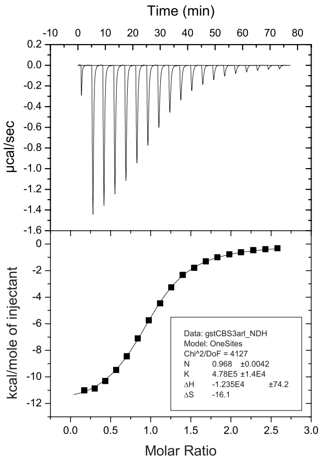

30 μM GST-CNNM3 299-452
300 μM ARL15 32-197

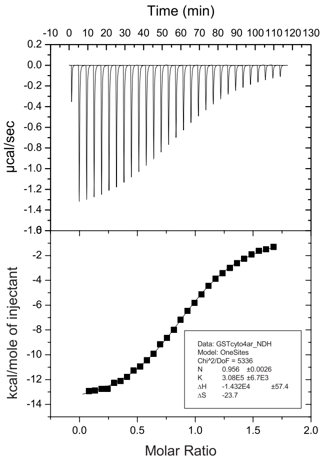

30 μM GST-CNNM4 356-726
300 μM ARL15 32-197

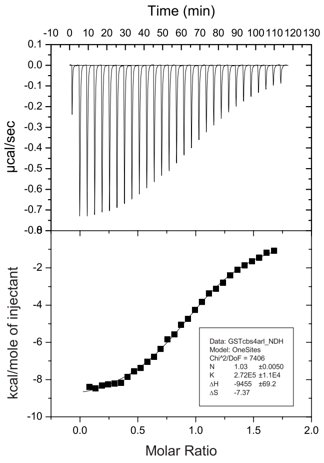

30 μM GST-CNNM4 356-511
300 μM ARL15 32-197

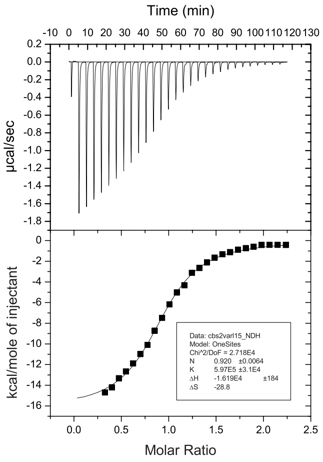

30 μM CNNM2 429-584
300 μM ARL15 32-197/GTP

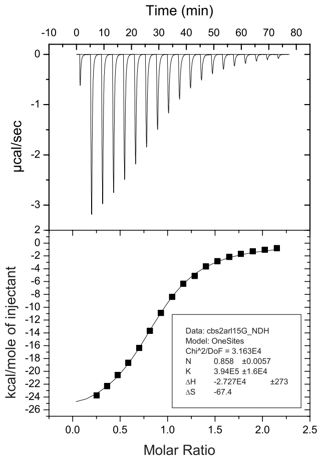

30 μM CNNM2 429-584
300 μM ARL15 32-197/GDP

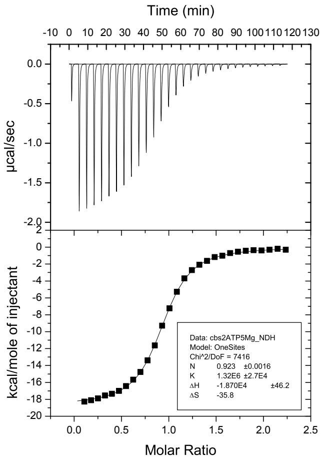

30 μM CNNM2 429-584
300 μM ARL15 32-197
In presence of 1 mM ATP and 5 mM $MgCl_2$

bioRxiv preprint doi: https://doi.org/10.1101/2023.01.19.524765; this version posted January 20, 2023. The copyright holder for this preprint (which was not certified by peer review) is the author/funder, who has granted bioRxiv a license to display the preprint in perpetuity. It is made available under aCC-BY 4.0 International license.

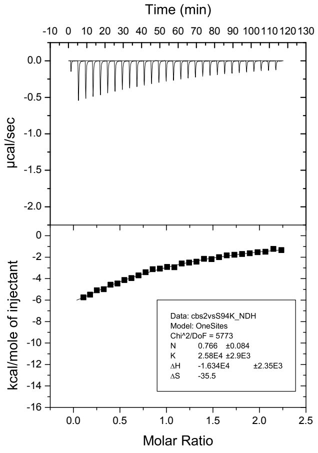

30 μM CNNM2 429-584
300 μM ARL15 32-197 **S94K**

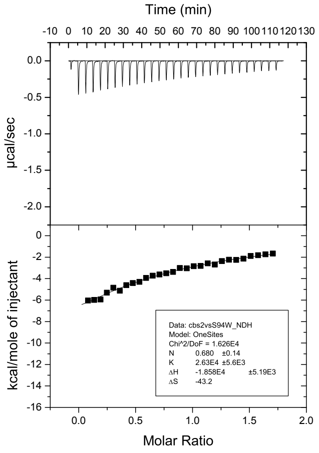

30 μM CNNM2 429-584
300 μM ARL15 32-197 **S94W**

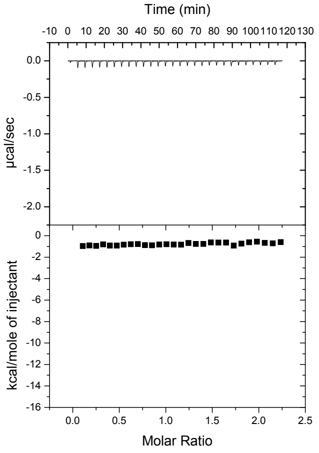

30 μM CNNM2 429-584
300 μM ARL15 32-197 **R95A**

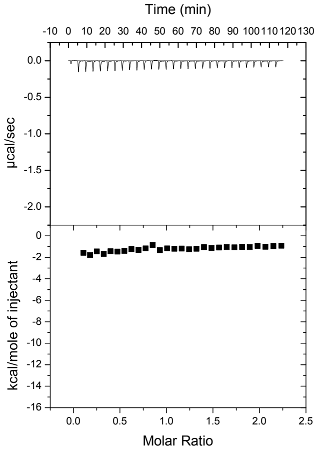

30 μM ARL15 32-197 **P130W**
300 μM CNNM2 429-584

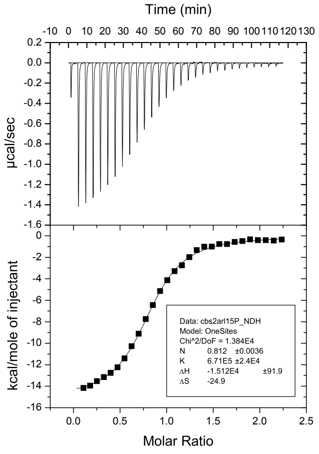

30 μM CNNM2 429-584
300 μM ARL15 32-197 **P163K**

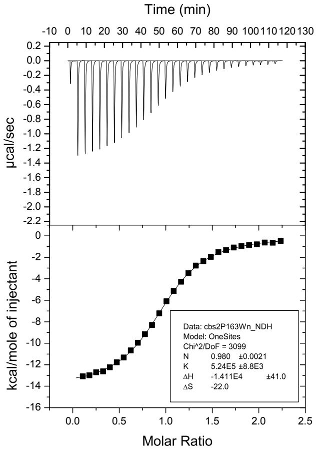

30 μM CNNM2 429-584
300 μM ARL15 32-197 **P163W**

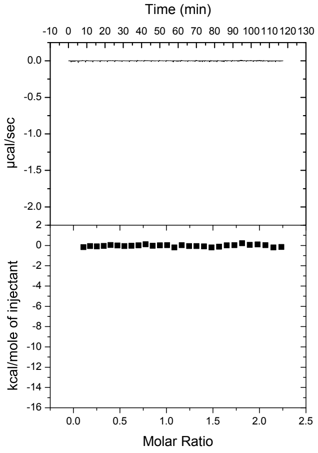

30 μM CNNM2 429-584 **H523K**
300 μM ARL15 32-197

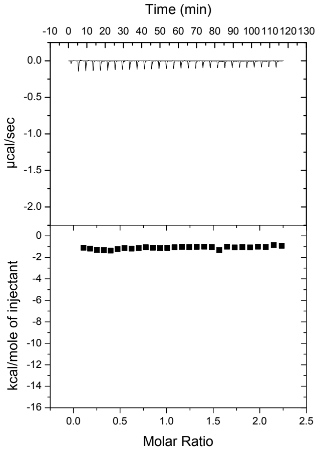

30 μM CNNM2 429-584 **F524K**
300 μM ARL15 32-197

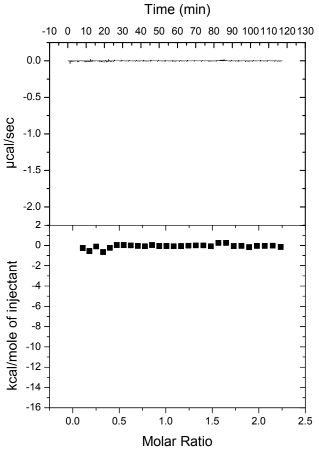

30 μM CNNM3 299-452 **H391K**
300 μM ARL15 32-197

bioRxiv preprint doi: https://doi.org/10.1101/2023.01.19.524765; this version posted January 20, 2023. The copyright holder for this preprint (which was not certified by peer review) is the author/funder, who has granted bioRxiv a license to display the preprint in perpetuity. It is made available under aCC-BY 4.0 International license.

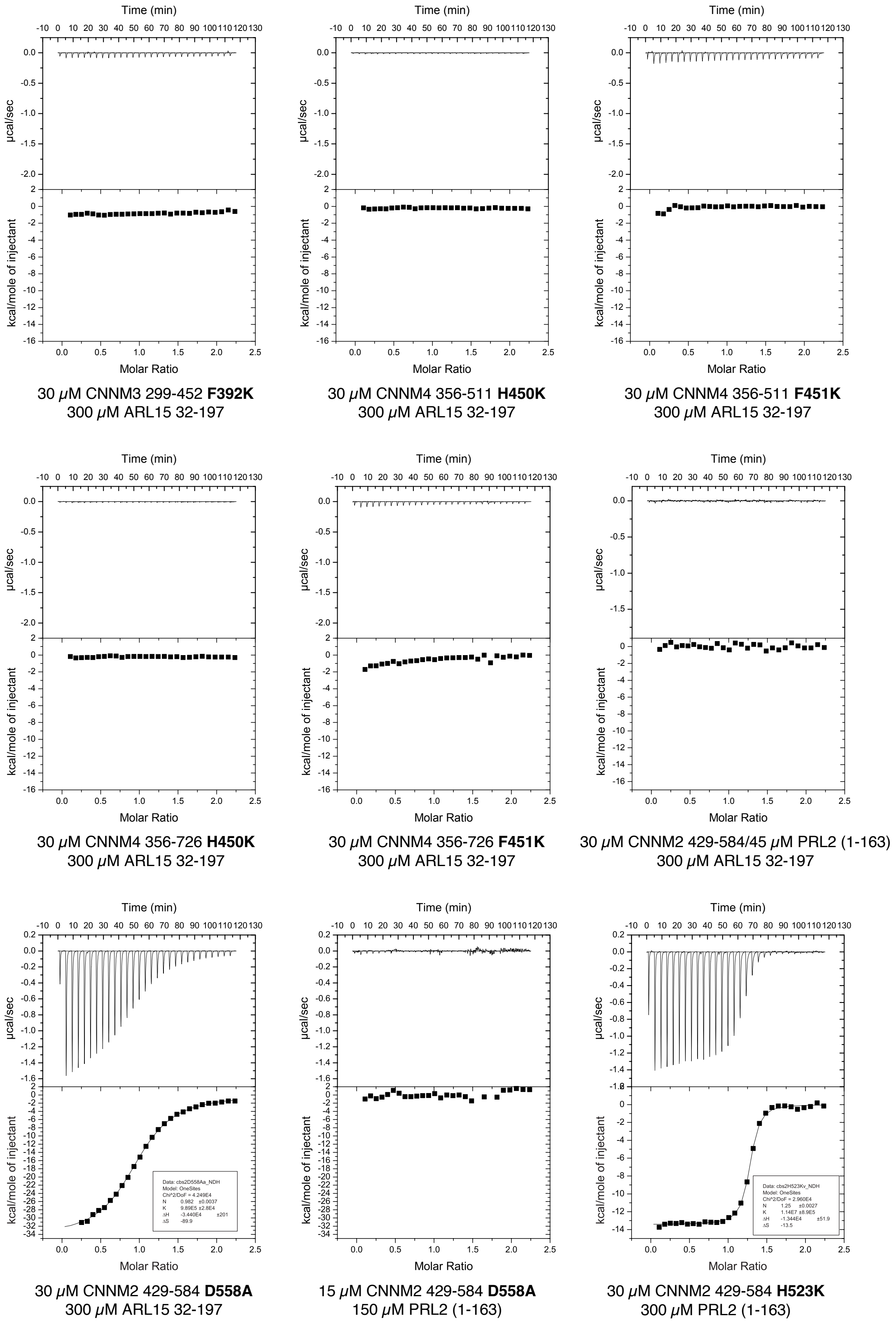

Supplemental Figure S1. ITC thermograms. Protein concentrations in the cell and the syringe are indicated.

bioRxiv preprint doi: https://doi.org/10.1101/2023.01.19.524765; this version posted January 20, 2023. The copyright holder for this preprint (which was not certified by peer review) is the author/funder, who has granted bioRxiv a license to display the preprint in perpetuity. It is made available under aCC-BY 4.0 International license.

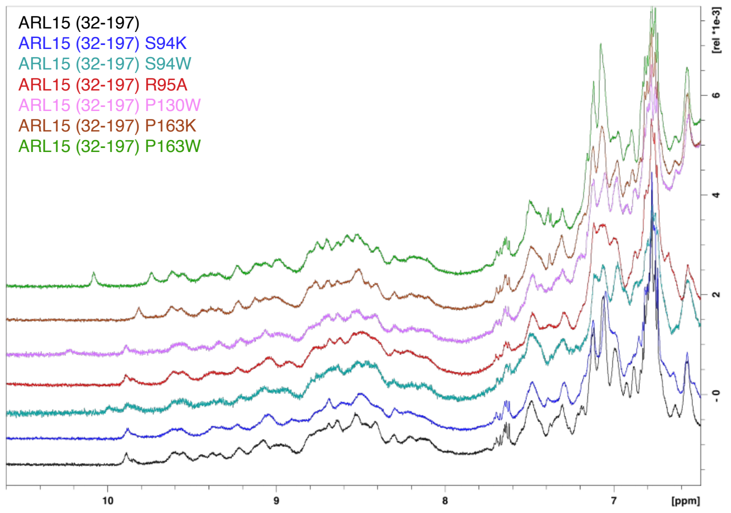

Supplemental Figure S2. Downfield ^{1}H NMR spectra of ARL15 GTPase domain (residue 32-197) and its mutants (S94K, S94W, R95A, P130W, P163K, P163W).

bioRxiv preprint doi: https://doi.org/10.1101/2023.01.19.524765; this version posted January 20, 2023. The copyright holder for this preprint (which was not certified by peer review) is the author/funder, who has granted bioRxiv a license to display the preprint in perpetuity. It is made available under aCC-BY 4.0 International license.

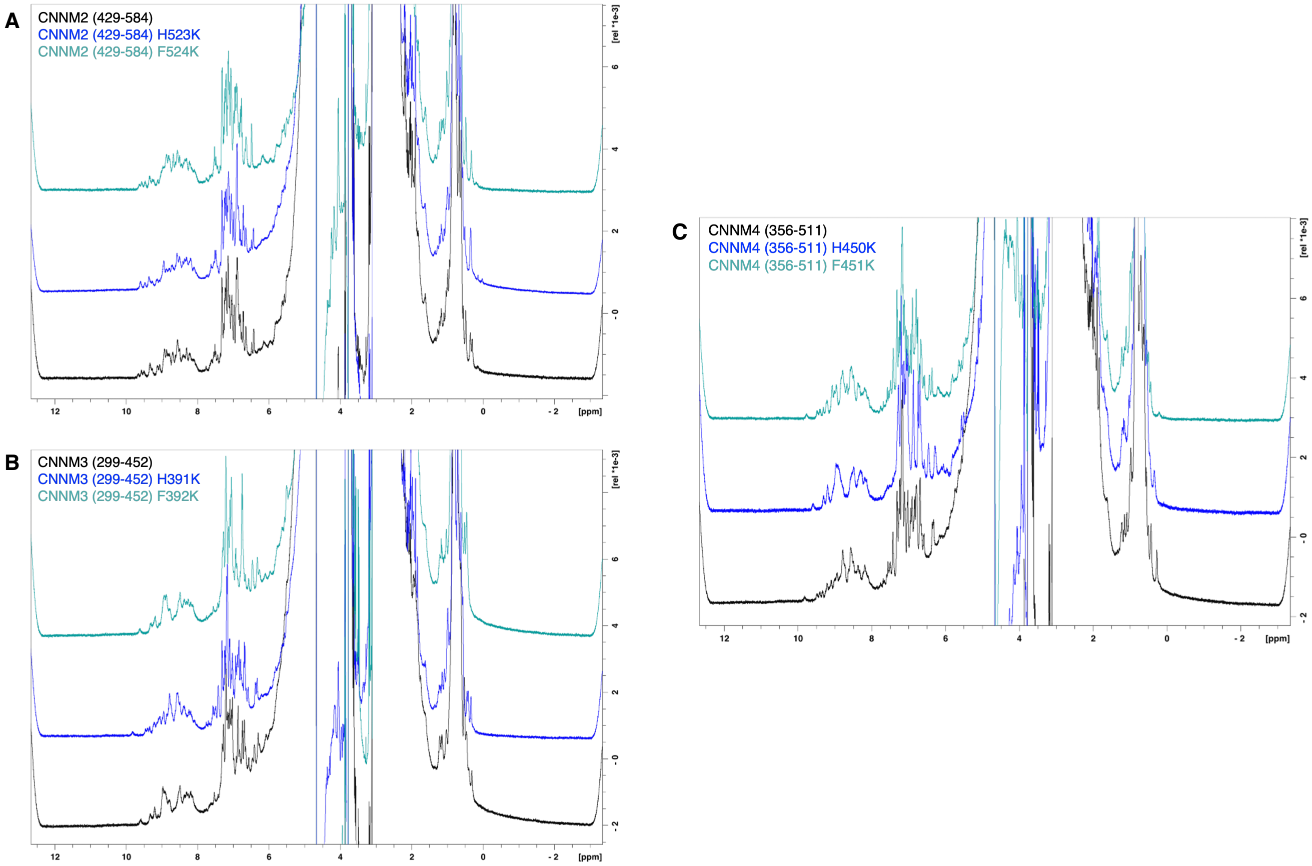

Supplemental Figure S3. ^{1}H NMR spectra of CNNM CBS-pair domains and their mutants. **A**, CNNM2 (429-584), CNNM2 (429-584) H523K, CNNM2 (429-584) F524K. **B**, CNNM3 (299-452), CNNM3 (299-452) H391K, CNNM3 (299-452) F392K. **C**, CNNM4 (356-511), CNNM4 (356-511) H450K, CNNM4 (356-511) F451K.

bioRxiv preprint doi: https://doi.org/10.1101/2023.01.19.524765; this version posted January 20, 2023. The copyright holder for this preprint (which was not certified by peer review) is the author/funder, who has granted bioRxiv a license to display the preprint in perpetuity. It is made available under aCC-BY 4.0 International license.

```
                                                G1 motif (P-loop)
                                                  GxxxxGKT
hARL15      MSDLRITEAFLYMDYLCFRALCCKGPPPARPEYDLVCIGLTGSGKTSLLSKLCSESPDNV   60
hARF1       ------------MGNIFA---NLFKGLFGKKEMRILMVGLDAAGKTTILYKLKLGEIVTT   45
hARF6       ----------------MG---KVLSKIFGNKEMRILMLGLDAAGKTTILYKLKLGQSVTT   41
hARL2       ------------MGLLTI---LKKMKQ-KERELRLLMLGLDNAGKTTILKKFNGEDIDTI   44
hARL3       ------------MGLLSI---LRKLKSAPDQEVRILLLGLDNAGKTTLLKQLASEDISHI   45
                                           *  :: :**  :***::* ::   .

        G2 motif (Switch I) G3 motif (Switch II)
             xTx                 DxxGQ
hARL15      VSTTGFSIKAVPFQNAILNVKELGGADNIRKYWSRYYQGSQGVIFVLDSASSEDDLEAAR   120
hARF1       IPTIGFNVETVEYKNISFTVWDVGGQDKIRPLWRHYFQNTQGLIFVVDSNDR-ERVNEAR   104
hARF6       IPTVGFNVETVTYKNVKFNVWDVGGQDKIRPLWRHYYTGTQGLIFVVDCADR-DRIDEAR   100
hARL2       SPTLGFNIKTLEHRGFKLNIWDVGGQKSLRSYWRNYFESTDGLIWVVDSADR-QRMQDCQ   103
hARL3       TPTQGFNIKSVQSQGFKLNVWDIGGQRKIRPYWKNYFENTDILIYVIDSADR-KRFEETG   104
              * **.::::  :.  :.: ::**  .:*  * .*: .:: :*:*:*. .  . .:

                                G4 motif                          G5 motif
                                 NKxD                              xAx
hARL15      NELHSALQHPQLCTLPFLILANHQDKPAARSVQEIKKYFELEPLARGKRWILQPCSLDDM   180
hARF1       EELMRMLAEDELRDAVLLVFANKQDLPNAMNAAEITDKLGLHSL-RHRNWYIQATCATSG   163
hARF6       QELHRIINDREMRDAIILIFANKQDLPDAMKPHEIQEKLGLTRI-RDRNWYVQPSCATSG   159
hARL2       RELQSLLVEERLAGATLLIFANKQDLPGALSSNAIREALELDSI-RSHHWCIQGCSAVTG   162
hARL3       QELAELLEEEKLSCVPVLIFANKQDLLTAAPASEIAEGLNLHTI-RDRVWQIQSCSALTG   163
            .**   : . .:    .*::**:**   *     * . : *  : * : * :*  .

hARL15      DALKDSFSQLINLLEEKDHEAVRM   204
hARF1       DGLYEGLDWLSNQLRNQK------   181
hARF6       DGLYEGLTWLTSNYKS--------   175
hARL2       ENLLPGIDWLLDDISSRIFTAD--   184
hARL3       EGVQDGMNWVCKNVNAKKK-----   182
            : :  .:  : .
```

Supplemental Figure S4. Sequence alignment of human ARL15 and other ARL/ARF GTPases. The glutamine residue that is unique to ARL15 is in the G3 motif.
